# Predicting above-ground density and distribution of small mammal prey species at large spatial scales

**DOI:** 10.1371/journal.pone.0177165

**Published:** 2017-05-17

**Authors:** Lucretia E. Olson, John R. Squires, Robert J. Oakleaf, Zachary P. Wallace, Patricia L. Kennedy

**Affiliations:** 1 Rocky Mountain Research Station, United States Department of Agriculture Forest Service, Missoula, Montana, United States of America; 2 Wyoming Game and Fish Department, Lander, Wyoming, United States of America; 3 Department of Fisheries and Wildlife and Eastern Oregon Agriculture & Natural Resource Program, Oregon State University, Union, Oregon, United States of America; University of Missouri Kansas City, UNITED STATES

## Abstract

Grassland and shrub-steppe ecosystems are increasingly threatened by anthropogenic activities. Loss of native habitats may negatively impact important small mammal prey species. Little information, however, is available on the impact of habitat variability on density of small mammal prey species at broad spatial scales. We examined the relationship between small mammal density and remotely-sensed environmental covariates in shrub-steppe and grassland ecosystems in Wyoming, USA. We sampled four sciurid and leporid species groups using line transect methods, and used hierarchical distance-sampling to model density in response to variation in vegetation, climate, topographic, and anthropogenic variables, while accounting for variation in detection probability. We created spatial predictions of each species’ density and distribution. Sciurid and leporid species exhibited mixed responses to vegetation, such that changes to native habitat will likely affect prey species differently. Density of white-tailed prairie dogs (*Cynomys leucurus*), Wyoming ground squirrels (*Urocitellus elegans*), and leporids correlated negatively with proportion of shrub or sagebrush cover and positively with herbaceous cover or bare ground, whereas least chipmunks showed a positive correlation with shrub cover and a negative correlation with herbaceous cover. Spatial predictions from our models provide a landscape-scale metric of above-ground prey density, which will facilitate the development of conservation plans for these taxa and their predators at spatial scales relevant to management.

## Introduction

Conversion of land from a natural state to one impacted by human activities affects more than half of Earth’s terrestrial habitat [1]. Greater than half of temperate grassland and savanna ecosystems are estimated to have been lost in North America [2], while sagebrush ecosystems occupy only 56% of their historic distribution in the United States [3]. Sagebrush steppe and prairie ecosystems are threatened due to anthropogenic land use changes such as increased urbanization, energy development, grazing of domestic animals, and conversion to cropland [4]. In addition, invasion by exotic annual grasses and encroachment of conifers threatens shrub-steppe ecosystems [3]. In the Great Basin ecoregion in the western United States, Rowland et al. [5] found that 55% of native sagebrush was at risk of replacement by non-native cheat grass (*Bromus tectorum*). Continued degradation of shrub-steppe and grassland ecosystems threatens wildlife species that depend on these habitats. Determining the impact of habitat loss on species at large spatial scales is both difficult and of great importance to conservation [6].

The choice of the most appropriate method to estimate population status at large spatial scales depends on both the information desired about the population, as well as the funding and time available for the research [7]. For example, occupancy analyses, which estimate the probability that one or more individuals of a species is present at a given location, have become a useful tool for conservation monitoring at large spatial scales [8]. Occurrence is often less expensive and intensive to measure than other state variables and is commonly used to estimate species distribution at large spatial scales [7,9,10]; however, abundance may be a more sensitive state variable for detecting population changes over time [11]. Abundance estimates rely on counts of individuals, and can be estimated with a variety of methods, using marked or unmarked individuals [12,13,14]. Abundance estimates are frequently more time-consuming and expensive to perform, but provide estimates of population abundance or density that may not be reflected in area of occupancy [9]. Due to the intensity of labor and cost, few studies have quantified abundance of animals at large spatial extents. When undertaken, however, large scale studies of abundance can provide valuable population information which may be unattainable from occurrence studies [15–17].

A decrease in abundance of prey can have a negative impact on the number or diversity of predators in an ecosystem [6,18,19]. Efforts to understand demography of predator species of concern at broad spatial extents would benefit from information on abundance of prey species; however, such information is typically limited to models of occurrence or indexes of relative abundance [20,21]. Small mammals are an important source of prey for a large number of sensitive or at risk raptor species in shrub-steppe and grassland ecosystems [22,23]; in Wyoming, Ferruginous hawks (*Buteo regalis*) and golden eagles (*Aquila chrysaetos*) depend on small mammals such as Wyoming ground squirrels (*Urocitellus elegans*), white-tailed prairie dogs (*Cynomys leucurus*), and leporid species for food [24], and burrowing owls (*Athene cunicularia*) require the burrows of small fossorial mammals for nesting [25]. Ferruginous hawks and burrowing owls are found only in shrub-steppe or grassland habitat, and are listed as sensitive species or birds of Conservation Concern, respectively, by the United States Fish and Wildlife Service, due primarily to habitat loss or alteration [25,26]. Raptor populations have been shown to respond to decreases in prey abundance with decreased productivity [27,28] or even local extinction [29].

Our objective was to estimate the influence of environmental covariates on landscape scale density and distribution of native sciurid and leporid species that were aboveground and thus available to predators in Wyoming, USA. The sage-steppe and grassland ecosystems of Wyoming have been increasingly degraded due to anthropogenic influences such as livestock grazing, agriculture, and energy development [4,30]. In Wyoming, energy development, in the form of oil and gas wells, has taken place almost exclusively in sagebrush and grassland habitats [31], and has more than doubled in number of wells since 2000 [32]. Since the loss and fragmentation of remaining sagebrush and grassland ecosystems is likely to continue, understanding how small mammal prey species might respond to these changes at a landscape scale has important conservation implications for these species, as well as the raptors and mammalian predators that depend on them. We surveyed small mammals over a large study area (114,217 km^2^) and used remotely sensed vegetation variables (including percent cover of sagebrush, herbaceous species, bare ground, and shrubs in general), biophysical variables (topography or climate), and anthropogenic features (petroleum wells and roads) to model spatially explicit estimates of above-ground density and distribution for four small mammal species groups. To accommodate imperfect detection and include environmental covariates correlated with density of each species group, we used a hierarchical distance-sampling model [33,34]. We used the best supported model for each species group to create spatial predictions of above-ground density and distribution over a broad spatial scale.

## Materials and methods

### Study area

**Fig 1:**
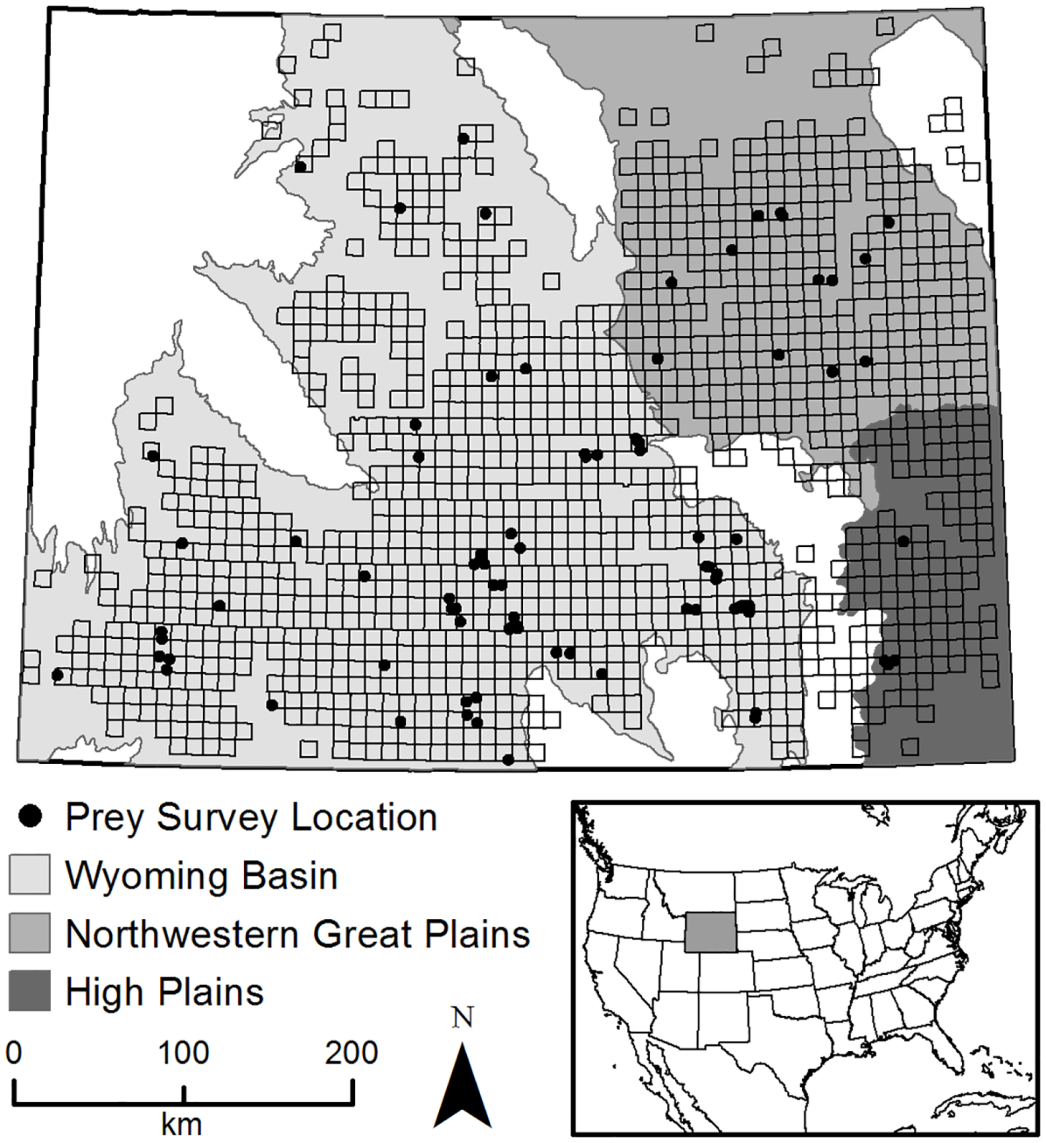
Locations surveyed for small mammal prey species in three non-mountainous ecoregions of Wyoming, 2010–2012. Survey locations are shown as block dots, small black squares show the extent of the randomly selected townships in the study area. Inset shows location of Wyoming in the United States. See text for more details on sampling design.

Our study area consisted of the shrub-steppe and grassland regions of Wyoming (Fig 1), within a 114,217 km^2^ area comprising the modeled distribution [21] of ferruginous hawks in Wyoming, since our study was conducted in conjunction with a larger study on ferruginous hawks [35–37] (see [36] for a detailed description of study area). Our study area was characterized by relatively low mean annual precipitation of 15 cm to 40 cm and elevation from approximately 1,000 m to 2,000 m [38]. Ecoregions within our study area included the sagebrush steppe of the Wyoming Basin (centroid 108.326° W, 42.560° N), the shortgrass and mixed-grass prairie of the High Plains (centroid 104.568° W, 41.929° N), and semiarid grasslands of the Northwestern Great Plains (centroid 105.610° W, 43.861° N), and excluded mountainous areas in the adjacent Central and Southern Rockies ecoregions [39]. All field work was carried out on public lands maintained by the Bureau of Land Management, U.S. Forest Service, and state of Wyoming, or on private land accessed with permission from landowners.

### Field methods

**Fig 2:**
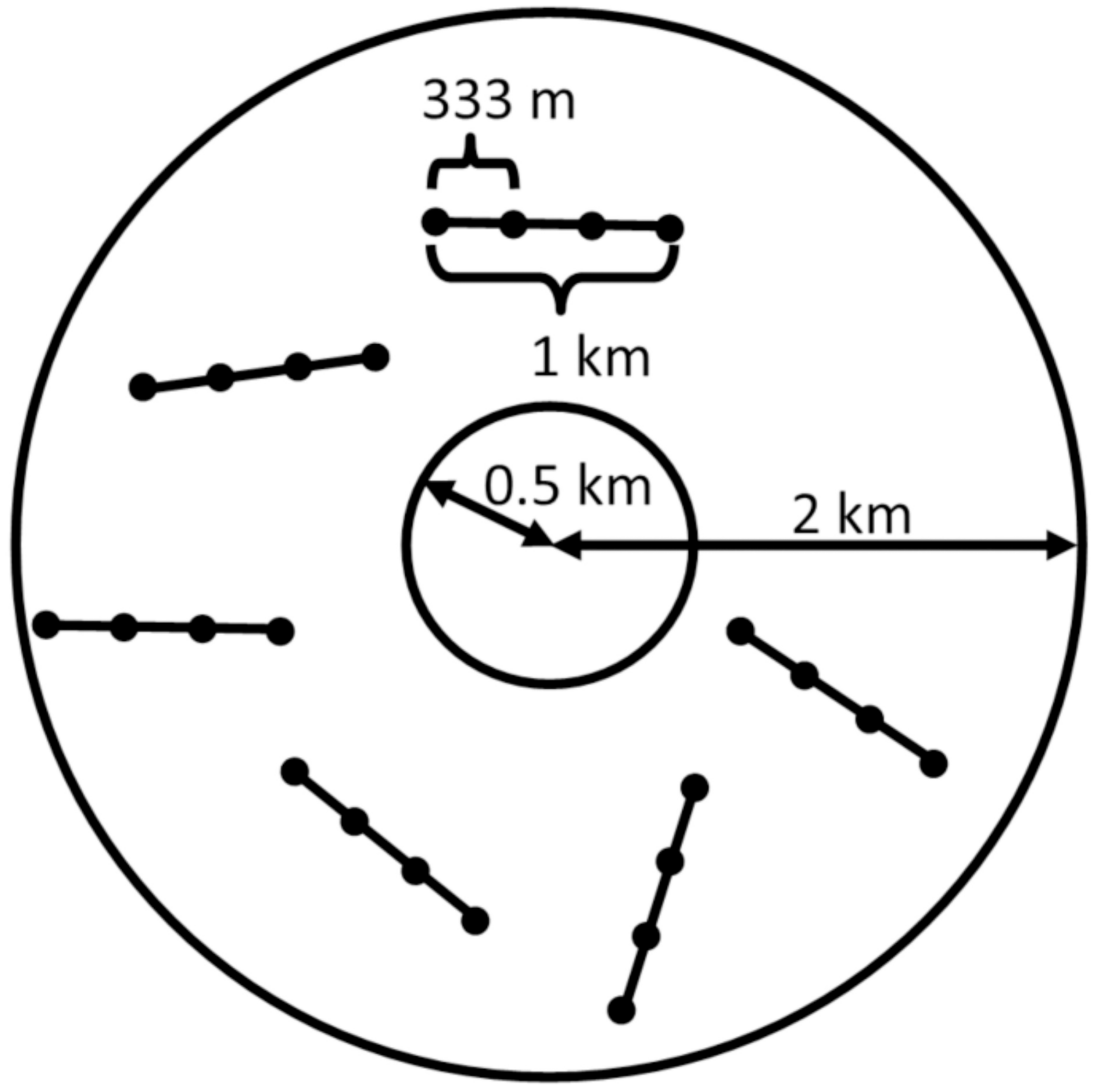
Example of the arrangement of sample points and transects within each survey site used to sample sciurid and leporid prey species in Wyoming, USA.

During the summers of 2010–2012, we conducted line-transect distance sampling of small mammals on nesting territories used by ferruginous hawks (n = 65) and random locations (n = 21) within our study area. We chose the line-transect method because: 1) it has been shown to perform equally well or better than other common methods such as mark-recapture or removal designs for estimating small mammal density [40], 2) it was less time-intensive than these methods, allowing us to sample an extremely large spatial extent during each field season, and 3) leporid species are known to hide until flushed and so detection is more likely using this method. A probabilistic sample of ferruginous hawk nest locations was obtained from a concurrent study in Wyoming [35–37] and prey sampling took place at these nests and random locations as part of an effort to model raptor prey abundance. Each survey site consisted of an annulus with an outer radius of 2 km and an inner radius of 0.5 km to avoid disturbing nesting ferruginous hawks. Within each annulus, we randomly sampled six line transects with four point transects spaced 333 m apart along each line (Fig 2). We sampled all survey sites on two occasions in 2011 and 2012, once in mid-May to early June and again in July to mid-August, and on one occasion in 2010. Surveys were conducted from approximately 0530 hr to 0900 hr each day. Point transect surveys consisted of slowly turning in a complete circle for 5 minutes while visually searching for sciurids. Observers measured the distance to each sciurid detected using a laser range finder. If sciurids were detected as a group, observers recorded the distance to the center of the group and the number of individuals. Leporid line transect surveys consisted of slowly walking the transect section and recording the species, distance, and azimuth to each leporid detected. In 2011 and 2012, we also recorded leporids encountered while walking between transects.

### Covariates and modeling approach

We considered a suite of vegetation, climate, topographic, and anthropogenic variables that we predicted would influence small mammal density or detectability (Table 1). We included high-resolution modeled vegetation variables since we hypothesized that small mammal density would likely be influenced by the presence of food or escape cover, both provided by vegetation [41,42]. Climate variables included winter and spring precipitation and temperature, which we hypothesized would influence primary productivity and thus, small mammal densities [43]. We hypothesized that elevation would relate to small mammal density at large spatial scales, while negative topographic position index and increased roughness would provide favorable conditions in the form of increased cover at a site level [44]. We predicted roads and energy development would reduce and fragment habitat, as well as potentially increase mortality via pest control or vehicle collision [45,46].

**Table 1:** Variables considered for use as covariates of small mammal prey density in the shrub-steppe and grassland regions of Wyoming, 2010–2012.

Type	Variable Name	Resolution	Description	Source
Vegetation	Bare	30 m	Estimated percent cover of bare ground	USGS Wyoming Sagebrush Products [47]
	Sage	30 m	Estimated percent cover of any sagebrush species	
	Shrub	30 m	Estimated percent cover of any shrub species	
	Herb	30 m	Estimated percent cover of herbaceous vegetation	
	Shrub Ht	30 m	Estimated shrub height (m)	
	Productivity	250 m	Annual normalized difference vegetation index (from 2010)	MODIS [48]
Climate	Ppt_Winter	4 km	Amount of precipitation in December and January	PRISM Climate Group, 2010, Oregon State University, (http://prism.oregonstate.edu)
	Ppt_Spring	4 km	Amount of precipitation in April and May	
	Temp_Spring	4 km	Average temperature in April and May	
	TMin_Winter	4 km	Minimum temperature in January	
	TMax_Summer	4 km	Maximum temperature in July	
Topographic	Elevation	30 m	Elevation (m)	USGS National elevation dataset
	TPI	30 m	Topographic position index (Index of ridges vs. drainages)	[49,50]
	Roughness	30 m	Ratio of 3-D surface area to 2-D surface area; index of surface roughness	[49,51]
Anthropogenic	Rd_Dist	1:4000	Distance to nearest road	BLM data (Wyoming BLM, unpublished data)
	Well_Dist	Vector Data	Distance to nearest oil or gas well	[32]
Sampling Occasion	OccYear-X		A 5-level factor variable indicating the year (Year: 2010, 2011, or 2012) and sampling occasion (X: 1 or 2) at which prey were sampled	Field data

For each variable type, we also evaluated two spatial extents: a fine scale of 250 m to capture conditions at a local level and a broad scale of 1000 m to capture conditions influencing density at a population or meta-population level. Due to differences in space use among study species, we chose these scales arbitrarily to represent a small and large spatial extent. We created each scale from rasters with a native resolution of 30 m using the Focal Statistics tool in ArcMap (ESRI 2011, Redlands, CA) to calculate the mean of all cells in a circular window with a radius of 250 m or 1000 m. We standardized each covariate by subtracting the mean of the raster from each cell and dividing by the standard deviation. Means and standard deviations of covariates are given in S1 Table in Supporting Information.

To avoid problems associated with sparse data and focus our analyses on the abundant prey species, we modeled density and distribution for three sciurid species with the most detections: white-tailed prairie dogs, least chipmunks (*Neotamias minimus*), and Wyoming ground squirrels. None of the leporid species were detected in large numbers, and observers had difficulty confidently identifying many leporids to species in the field; thus, to achieve sufficient sample sizes for modeling, we grouped white-tailed jackrabbits (*Lepus townsendii*), desert cottontails (*Sylvilagus audobonii*), and unknown leporids into a single model representing abundance of leporids available to predators. A small number of Black-tailed jackrabbits (*Lepus californicus*; N = 2), eastern cottontails (*Sylvilagus floridanus*; N = 2), and pygmy rabbits (*Brachylagus idahoensis*; N = 5) identified during surveys were also included in this group. We created models specific to each species group based on information on habitat associations and range maps from the literature. Both white-tailed jackrabbits and desert cottontails have statewide distributions, and are found in relatively similar habitats [52,53], allowing them to be addressed with a single model encompassing all three ecoregions in the study area. Least chipmunks also have a statewide distribution [54]. Wyoming ground squirrels are limited to the southern half of Wyoming [55] and white-tailed prairie dogs occur in the central and western parts of the state [56]. Thus, we modeled the density of Wyoming ground squirrels only in the southern half of the Wyoming Basin and the High Plains ecoregions, and white-tailed prairie dogs in the Wyoming Basin. For each species group, we did not include transects from areas that were outside of a species’ known range, nor did we make spatial predictions of density in these areas.

To model density and detection, we used the ‘distsamp’ function in program R [57] package ‘unmarked’ [58], which is based on the hierarchical distance-sampling model of Royle et al. [33]. The ‘distsamp’ function jointly estimates two parameters: abundance (λ), which treats the counts of individuals as a Poisson distributed variable, and detection (*p*), which models detection probability based on the distances recorded to each individual using the specified detection function; both components are allowed to vary in response to environmental covariates [33,34]. To determine whether our sampling scheme would necessitate separate abundance models for prey on hawk territories versus random locations, we initially considered the presence or absence of a nearby ferruginous hawk nest as a covariate for abundance. Our exploratory analysis of this variable in a univariate model indicated that hawk nest presence or absence was not a strong predictor of prey abundance because nest presence consistently ranked near or below the null (i.e., intercept-only) model when compared with AIC_c_ (Akaike Information Criteria for finite sample sizes [59]). Therefore, we did not include this variable in further analyses and pooled data from hawk territories and random locations. We truncated point transect data by 10% and line transect data by 5% to avoid a long-tailed distribution, as recommended in Buckland et al. [60]. To determine the best-fitting detection function for each species group, we initially modeled each sampling occasion separately, fitting both hazard and half-normal detection functions, and selected the best fitting function with AIC_c_. We retained the detection function that was the most frequently best-fitting over all occasions for each prey species group for use in future models.

Our modeling process consisted of two steps: an initial covariate screening process, followed by a model selection process. First, to select covariates for use in later candidate models, we tested univariate models for both spatial scales in turn with each covariate as a predictor on the abundance parameter while holding the detection parameter constant, and then on the detection parameter while holding the abundance parameter constant. To avoid using correlated variables and to reduce the number of potential covariates in the model, we retained only those covariates that ranked better than or equal to the null model for abundance. Only a single top performing covariate for detection was considered, due to model computation time. We screened all variables for pairwise multicollinearity using Pearson’s r > |0.7|; if variables were correlated, we retained the variable with the lower univariate AIC_c_ score.

Second, we used the selected covariates to create additive, multivariate abundance and detection models for each species group. As a final check for multicollinearity, we calculated the variance inflation factor (VIF) for each species’ global abundance model; if multicollinearity was present (VIF > 4) we removed covariates with the most collinearity from the global model [61]. We considered six candidate abundance models representing different categories of covariates: a global model, a vegetation-only model, a topographic-only model, a climate-only model, an anthropogenic-only model, and a model in which only the top performing covariate in each category was included. For detection, we considered four candidate detection models: a continuous version of the top-performing covariate from step 1, a categorical version of this covariate that binned the top-performing detection covariate data into three equal intervals based on minimum and maximum values, a categorical occasion covariate with 5 levels to account for differences in detection over all 5 sampling occasions, and an additive combination of both occasion and the categorical detection covariate.

We first evaluated the detection models while holding abundance constant, and chose the top performing detection model using AIC_c_. We then used the top-performing detection model to compare the six candidate abundance models and selected the top abundance model structure using AIC_c_. Finally, we reran the top-performing abundance model with all previous detection models to verify that the best detection model had not changed after adding structure to the abundance parameter. We did not assume populations were subject to demographic closure between sampling occasions due to the occurrence during the survey season of deaths, births, and emergence of young above ground. Thus, we treated each sampling occasion (n = 5) as an independent event and included an indicator covariate for sampling occasion in all abundance models during the model selection process to accommodate potential differences in density among occasions.

We used the ‘predict’ function in package ‘unmarked’ to estimate occasion-specific densities and standard errors at each sampled location for the top-performing model for each species group. If model fit statistics indicated over-dispersion, we estimated an over-dispersion parameter ($\hat{c}$) for the global model and used this to inflate standard errors by $\sqrt{\hat{c}}$ as well as calculate QAIC_c_ (quasi-Akaike Information Criteria) for model selection, as recommended by Burnham & Anderson [62]. We used the adjusted standard errors to compute confidence intervals for density and detection beta coefficients, and used a nonparametric bootstrap procedure to estimate occasion-and year-specific density estimates and standard errors for over-dispersed models. We averaged these estimates over all sampled locations to provide a general estimate of prey density per sampling occasion.

We used modeled beta coefficients to generate spatial predictions of density for each species group over their distribution within the study area. Extrapolating density estimates outside of the range of sampled covariates can lead to erroneous predictions [63]. To decrease potential error in out-of-sample predictions, we constrained covariate values outside the sampling frame to the range of values observed within the sampled areas for each species group.

### Model evaluation

We evaluated our top performing models for each species group using bootstrap procedures. We used the ‘parboot’ function from package ‘unmarked’ in R to simulate 1000 new datasets from our fitted top model; at each iteration, we refitted the model and estimated the sum of squared errors (SSE) from the model predictions. We then compared the SSE from the model fit on our original dataset to the distribution of SSEs from the bootstrap procedure using a Freeman-Tukey fit statistic [34]. We considered the fit of a model to be good when its SSE did not differ significantly from the bootstrap distribution, indicating the model fit the underlying distribution of the data and was not over- or under-dispersed.

## Results

We detected a total of 12 sciurid and leporid species during surveys in 2010–2012: black-tailed jackrabbit, white-tailed jackrabbit, desert cottontail, eastern cottontail, pygmy rabbit, white-tailed prairie dog, black-tailed prairie dog (*Cynomys ludovicianus*), Wyoming ground squirrel, Uinta ground squirrel (*Urocitellus armatus*), thirteen-lined ground squirrel (*Ictidomys tridecemlineatus*), least chipmunk, and Uinta chipmunk (*N*. *umbrinus*; for all nomenclature authority refer to Bradley et al. [64]). Most species were rarely detected; majority of detections (> 95%) comprised Wyoming ground squirrels and white-tailed prairie dogs. We counted 14,227 white-tailed prairie dogs, 348 least chipmunks, 3,772 Wyoming ground squirrels, and 382 leporids (Table 2). In total, we sampled 86 sites, with 65 near ferruginous hawk nests and 21 in randomly chosen locations.

**Table 2:** Number of individuals of the most abundant small mammal species groups detected during each sampling occasion and summed over all sampling occasions from line transect surveys in Wyoming, 2010–2012.

Year	2010	2011		2012		Total
Sampling Occasion	1	1	2	1	2	
White-tailed prairie dogs	2,888	3,535	3,267	2,723	1,814	14,227
Least chipmunks	24	79	99	65	81	348
Wyoming ground squirrels	468	1,788	731	623	162	3,772
Leporids	49	42	66	111	114	382

The leporid group represents several pooled leporid species; see text for details.

### Density and detection probability

We truncated observations to 273 m for white-tailed prairie dogs, 177 m for least chipmunks, 240 m for Wyoming ground squirrels, and 60 m for leporids. Based on individual distance analyses, we selected the half-normal detection function for white-tailed prairie dogs, least chipmunks, and Wyoming ground squirrels, and the hazard-rate function for leporids due to their spiked distribution [60,65]. Detection probabilities for each species group over all sampling occasions were 0.37 (95% CI: 0.33–0.40) for white-tailed prairie dogs, 0.19 (95% CI: 0.14–0.25) for least chipmunks, 0.30 (95% CI: 0.27–0.34) for Wyoming ground squirrels, and 0.24 (95% CI: 0.14–0.41) for leporids.

Average above-ground densities over all sites varied among species groups and among sampling occasions within species groups (Table 3). White-tailed prairie dogs averaged 19.7 individuals per km^2^ (SD = 56.0) with a range of 11.1–28.7 per km^2^ over all occasions. Least chipmunks had an average density of 2.0 per km^2^ (SD = 1.4) and a range of 1.2–2.5 per km^2^ over all sampling occasions. Wyoming ground squirrels had an average above-ground density of 8.7 per km^2^ (SD = 9.6) over all sampling occasions, with a range of 2.1–20.6 per km^2^ among sampling occasions. Leporids had an average density of 3.4 per km^2^ (SD = 3.0) and a range of 2.1–4.6 per km^2^ over all occasions.

**Table 3:** Occasion-specific density estimates (individuals/km^2^; standard deviation in parentheses) averaged over all sampled sites for the most abundant small mammal species groups modeled from line transect surveys in Wyoming, 2010–2012.

Year	2010	2011	2011	2011	2012	2012	2012	All Years
Sampling Occasion	1	1	2	Avg	1	2	Avg	
White-tailed prairie dogs	28.7 (113.4)	21.0 (52.4)	16.7 (43.4)	18.9 (48.1)	21.2 (48.6)	11.1 (22.2)	16.0 (36.5)	19.7 (56.0)
Least chipmunks	1.2 (0.7)	2.3 (1.5)	2.5 (1.6)	2.4 (1.6)	2.2 (1.5)	1.6 (1.0)	1.9 (1.3)	2.0 (1.4)
Wyoming ground squirrels	6.7 (3.5)	20.6 (13.5)	5.7 (3.6)	13.3 (12.5)	7.1 (4.7)	2.1 (1.4)	4.5 (4.2)	8.7 (9.6)
Leporids	4.1 (1.3)	2.1 (0.7)	3.1 (0.9)	2.6 (2.3)	3.8 (1.1)	4.6 (1.3)	4.2 (3.6)	3.4 (3.0)

Estimated densities were averaged over all sites and occasions to generate the ‘All Years’ density.

### Environmental covariates

Univariate analyses reduced the number of covariates in each species group model to between seven and nine (Table 4). For all species groups except least chipmunks, the global model was the best supported model of abundance, with no model uncertainty (Table 5). For least chipmunks, the top supported model was the vegetation-only model, although the global model was within 2 ΔAIC_c_. Based on model weight, the vegetation-only model was 2.06 times more likely to be the best explanation for least chipmunk density, and the small change in AIC_c_ between the vegetation model and the global model indicated that the four additional covariates in the global model were uninformative [66].

**Table 4:** Global abundance and top-performing detection model structures for each small mammal species group surveyed in Wyoming, 2010–2012.

Group	Model Structure
White-tailed prairie dogs	*p(Shrub*_*1000*_ *category +Occasion) λ(Shrub*_*1000*_*+Herb*_*1000*_*+ Temp_Spring+Ppt_Spring+Ppt_Winter+TPI*_*1000*_*+ Well_Dist+Occasion)*
Least chipmunks	*p(Occasion) λ(Shrub*_*1000*_*+Shrub_Ht*_*1000*_*+Herb*_*1000*_*+Ppt_Spring+Ppt_Winter+Elevation*_*1000*_*+ Rd_Dist+Occasion)*
Wyoming ground squirrels	*p(Bare*_*1000*_ *category +Occasion) λ(Sage*_*1000*_*+Bare*_*1000*_*+Shrub_Ht*_*1000*_*+TMin_Winter+Ppt_Winter+Roughness*_*250*_*+ Elevation*_*1000*_*+Well_Dist+Rd_Dist+Occasion)*
Leporids	*p(Sage*_*1000*_*) λ(Bare*_*1000*_*+Sage*_*1000*_*+Shrub_Ht*_*250*_*+Ppt_Winter+TMin_Winter+Tmax_Summer+ Roughness*_*1000*_*+Well_Dist+Occasion)*

The top-performing detection model (*p*) was used with all abundance models during multivariate model selection, while the covariates in the global abundance model, *λ*, were used to create six candidate abundance models (see text for details). The scale at which each variable was selected, either 250 m or 1000 m, is given in subscript. Covariates are defined in Table 1.

**Table 5:** Abundance model selection results for small mammal species groups surveyed in the shrub-steppe and grassland regions of Wyoming, 2010–2012.

Species	Model	K	AIC_c_	ΔAIC_c_	AIC_c_ Wt	LL
White-tailed prairie dogs*	Global	20	27897.77	0	1	-13928.83
	Top Mix	17	28038.82	141.04	0	-14002.37
	Anthropogenic	14	29144.10	1246.33	0	-14558.02
	Climate	16	29249.32	1351.54	0	-14608.62
	Vegetation	14	29292.47	1394.70	0	-14631.20
	Topography	15	30308.66	2410.89	0	-15140.30
	Null	9	30520.86	2623.09	0	-15251.42
Least chipmunks	Vegetation	10	3984.91	0	0.66	-1982.44
	Global	14	3986.37	1.46	0.32	-1979.16
	Top Mix	11	3992.47	7.56	0.02	-1985.22
	Topography	8	4009.68	24.77	0	-1996.83
	Climate	9	4021.27	36.36	0	-2001.62
	Anthropogenic	8	4032.20	47.29	0	-2008.09
	Null	6	4128.01	143.10	0	-2058.00
Wyoming ground squirrels*	Global	22	17763.84	0.00	1	-8859.85
	Topography	15	18009.13	245.29	0	-8989.53
	Top Mix	17	18054.07	290.23	0	-9009.99
	Vegetation	16	18300.84	537.00	0	-9134.38
	Anthropogenic	15	18474.89	711.05	0	-9222.41
	Climate	15	18503.50	739.67	0	-9236.72
	Null	13	18582.54	818.70	0	-9278.25
Leporids	Global	16	2775.86	0	1	-1371.85
	Top Mix	12	2791.69	15.84	0	-1383.80
	Topography	9	2802.00	26.14	0	-1391.97
	Climate	11	2814.39	38.53	0	-1396.16
	Vegetation	11	2815.02	39.17	0	-1396.47
	Anthropogenic	9	2816.50	40.64	0	-1399.22
	Null	6	2908.28	132.42	0	-1448.13

Model, the abundance model description (see text for details); K, number of model parameters; AIC_c_ Wt, AIC_c_ Weight; LL, log likelihood. Species groups marked with an * indicate those that used QAIC_c_ for model selection.

**Fig 3:**
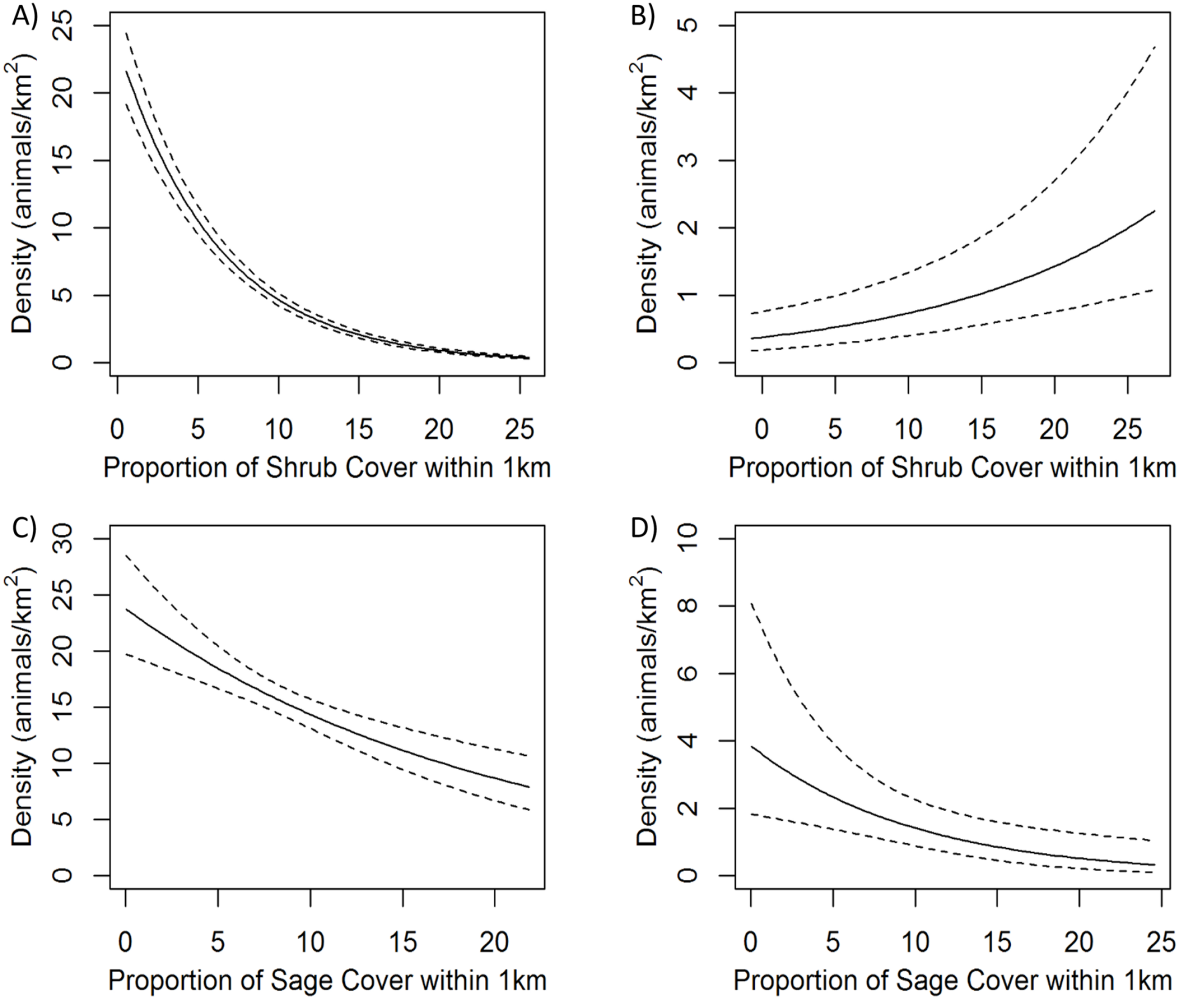
Modeled response of density (individuals/km^2^) of small mammal species groups to proportion of shrub or sagebrush cover. Top panels show proportion of shrub cover, bottom panels show proportion of sagebrush cover; dotted lines show 95% confidence intervals. Panel A) shows white-tailed prairie dogs, B) least chipmunks, C) Wyoming ground squirrels, and D) leporids (*Sylvilagus* sp. and *Lepus townsendii*).

**Fig 4:**
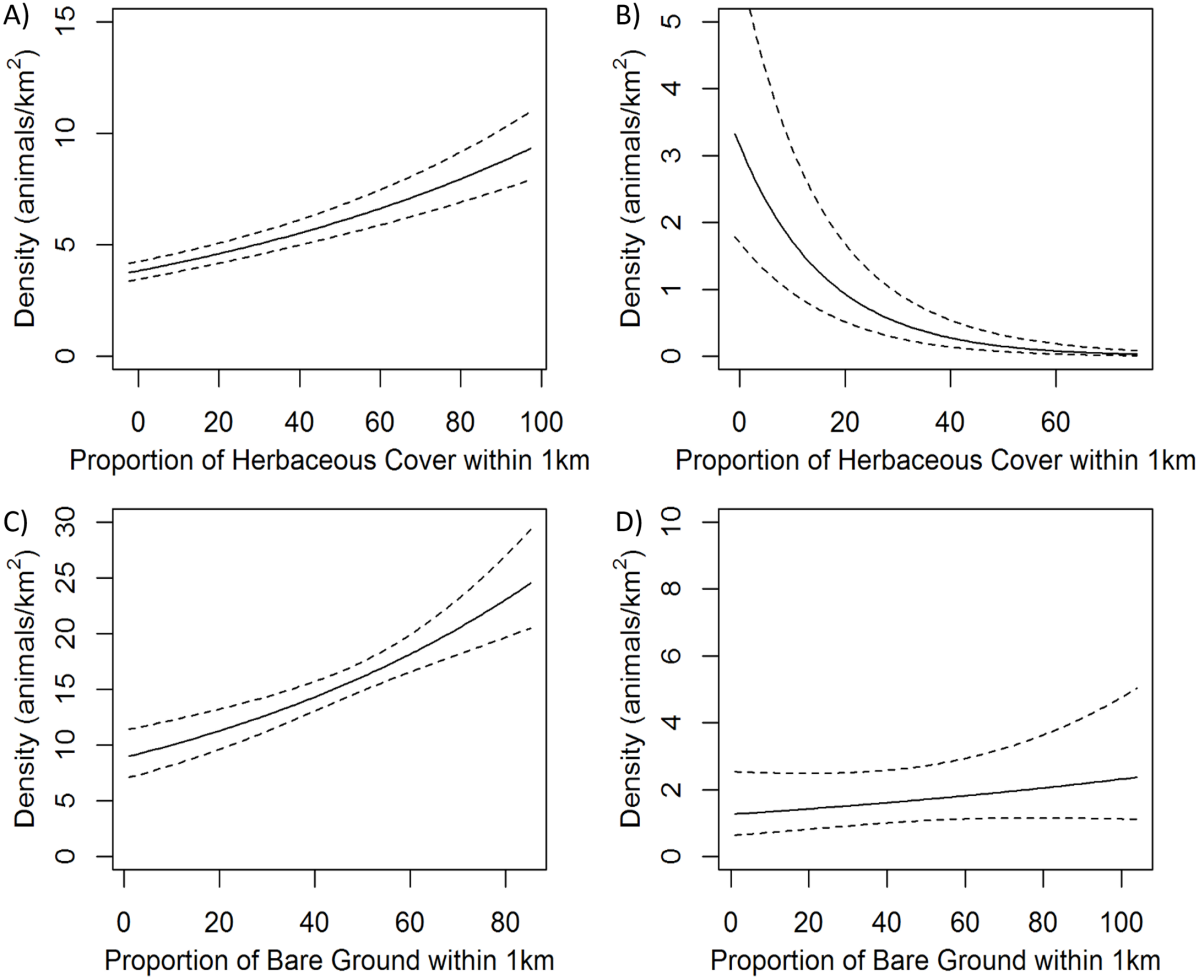
Modeled response of density (individuals/km^2^) of small mammal species groups to proportion of herbaceous cover or bare ground. Top panels show herbaceous cover, bottom panels show bare ground; dotted lines show 95% confidence interval. Panel A) shows white-tailed prairie dogs, B) least chipmunks, C) Wyoming ground squirrels, and D) leporids (*Sylvilagus* sp. and *Lepus townsendii*).

**Fig 5:**
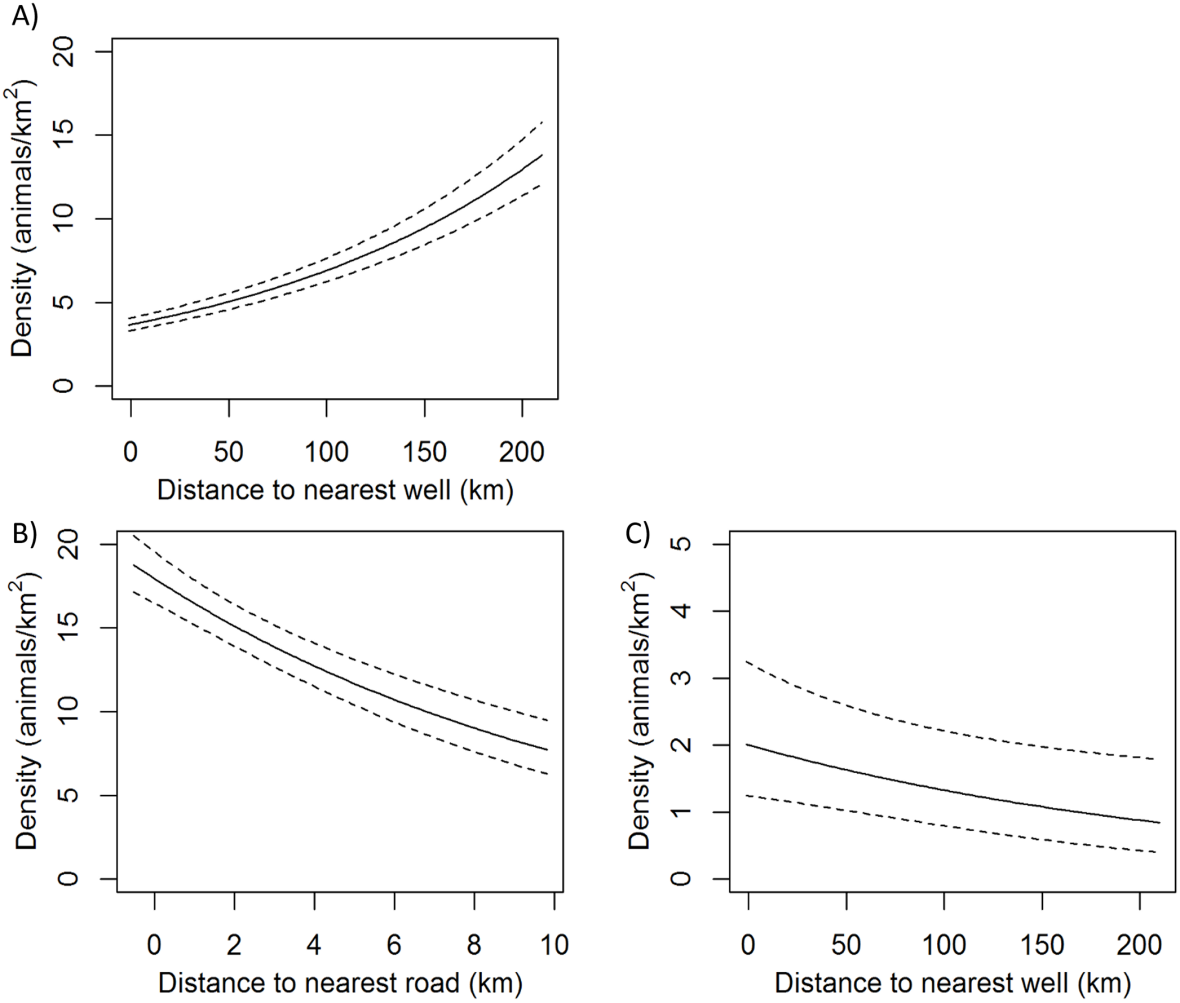
Modeled response of density (individuals/km^2^) of small mammal species groups to anthropogenic covariates: distance to nearest oil/gas well or distance to nearest road. Dotted lines show 95% confidence interval. Panel A) shows white-tailed prairie dogs, B) Wyoming ground squirrels, and C) leporids (*Sylvilagus* sp. and *Lepus townsendii*). Least chipmunks did not respond significantly to anthropogenic covariates.

**Fig 6:**
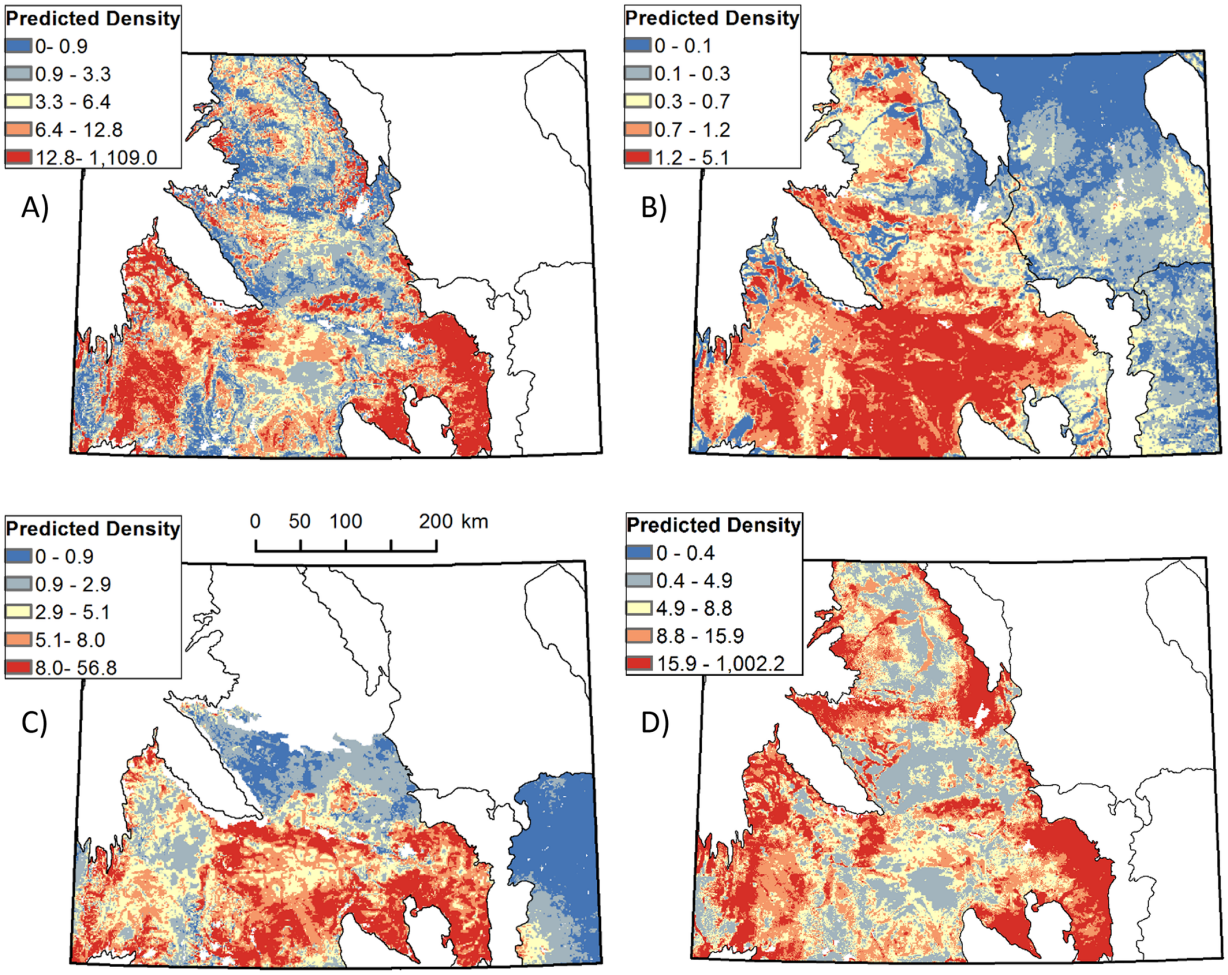
Spatial predictions of low to high density for each of the four small mammal species groups: A) white-tailed prairie dogs, B) least chipmunks, C) Wyoming ground squirrels, and D) leporids (*Sylvilagus* sp. and *Lepus townsendii*). Cool colors indicate lower density, warm colors higher. White areas in each map show mountainous ecoregions which were not included in the study area.

Vegetation covariates were significant predictors of above-ground density for all four species groups and variables representing topography, climate, and anthropogenic factors were significantly related to density for all except least chipmunks (based on coefficient 95% CI overlap with 0; Table 6, Figs 3–5). White-tailed prairie dog density decreased with percent cover of shrubs, higher average spring temperature, and areas with positive topographic position index (indicating slopes or ridges), and increased with herbaceous cover, greater winter precipitation, and distance from oil and gas wells. Least chipmunk density increased with greater shrub cover and decreased in response to greater herbaceous cover. Wyoming ground squirrel density decreased with increasing percent cover of sagebrush and greater topographic roughness, and increased with proportion of bare ground, shrub height, elevation, warmer minimum winter temperatures, and proximity to roads. Leporid density was greatest in areas with lower proportions of sagebrush cover, greater topographic roughness, lower minimum winter temperature, and increased proximity to oil and gas wells. The 1000-m scale was more predictive for all covariates in all species group models except for shrub height, which performed better at the 250-m scale for leporids, and surface roughness, which performed better at the 250-m scale for Wyoming ground squirrels. Density for all species groups was significantly different for at least two of five sampling occasions compared to the first sampling occasion (Table 6), but there was no consistent pattern in these differences among species groups (S1 Fig). Spatial density predictions for each species group are shown in Fig 6.

**Table 6:** Standardized beta coefficients for the top performing distance model for each modeled small mammal species group from line transect surveys in Wyoming, 2010–2012.

	White-tailed prairie dogs	Least chipmunks	Wyoming ground squirrels	Leporids
Abundance Covariates				
Herb_1000_	**0.13 (0.10 to 0.16)**	**-0.89 (-1.09 to -0.7)**		
Bare_1000_			**0.22 (0.14 to 0.31)**	0.11 (-0.09 to 0.31)
Shrub_1000_	**-0.57 (-0.61 to -0.52)**	**0.28 (0.16 to 0.41)**		
Sage_1000_			**-0.20 (-0.28 to -0.11)**	**-0.39 (-0.65 to -0.13)**
Shrub Ht_1000_		-0.12 (-0.27 to 0.03)	**0.33 (0.28 to 0.38)**	
Shrub Ht_250_				0.02 (-0.14 to 0.18)
Ppt_Spring	0.02 (-0.01 to 0.05)			
Ppt_Winter	**0.17 (0.14 to 0.21)**		0.00 (-0.04 to 0.05)	-0.10 (-0.23 to 0.03)
Temp_Spring	**-0.35 (-0.38 to -0.31)**			
TMin_Winter			**0.17 (0.12 to 0.22)**	**-0.15 (-0.28 to -0.02)**
TMax_Summer				0.08 (-0.06 to 0.23)
Elevation_1000_			**0.63 (0.57 to 0.69)**	
Roughness_250_			**-0.37 (-0.43 to -0.31)**	
Roughness_1000_				**0.26 (0.16 to 0.36)**
TPI_1000_	**-0.10 (-0.14 to -0.07)**			
Well_Dist	**0.33 (0.30 to 0.35)**		-0.02 (-0.06 to 0.01)	**-0.17 (-0.32 to -0.03)**
Rd_Dist			**-0.18 (-0.22 to -0.13)**	
Occ2011-1	**-0.29 (-0.41 to -0.14)**	**0.84 (0.17 to 1.51)**	**1.17 (1.01 to 1.33)**	**-0.84 (-1.31 to -0.38)**
Occ2011-2	**-0.45 (-0.58 to -0.30)**	**0.92 (0.26 to 1.58)**	-0.14 (-0.34 to 0.05)	**-0.47 (-0.9 to -0.03)**
Occ2012-1	**-0.58 (-0.72 to -0.41)**	**0.84 (0.14 to 1.53)**	0.15 (-0.04 to 0.35)	-0.29 (-0.71 to 0.14)
Occ2012-2	**-1.19 (-1.36 to -1.00)**	0.44 (-0.24 to 1.12)	**-1.08 (-1.36 to -0.8)**	-0.10 (-0.52 to 0.31)
Detection Covariates				
Shrub_1000_.M	**-0.10 (-0.14 to -0.05**)			
Shrub_1000_.H	**-0.28 (-0.40 to -0.13)**			
Sage_1000_				**0.25 (0.02 to 0.48)**
Bare_1000_.M			**0.20 (0.12 to 0.27)**	
Bare_1000_.H			**0.22 (0.13 to 0.3)**	
Occ2011-1	**0.14 (0.08 to 0.22)**	0.07 (-0.18 to 0.32)	-0.05 (-0.12 to 0.02)	
Occ2011-2	**0.26 (0.18 to 0.34)**	0.13 (-0.12 to 0.38)	**0.19 (0.09 to 0.28)**	
Occ2012-1	0.05 (-0.02 to 0.13)	0.01 (-0.25 to 0.27)	0.02 (-0.06 to 0.11)	
Occ2012-2	**0.16 (0.07 to 0.27)**	**0.33 (0.05 to 0.6)**	-0.07 (-0.18 to 0.05)	

Coefficients for both abundance and detection parameters are given with 95% CI in parentheses and significant predictors shown in bold. The scale at which covariates were included is given as subscript after the variable name; categorical detection covariates are given an M or H to indicate Medium or High factor level, respectively, compared to the Low reference group. Covariates are defined in Table 1.

Sampling occasion was supported as a detection covariate for white-tailed prairie dogs, least chipmunks, and Wyoming ground squirrels; however, only one occasion out of five was significantly different in detection probability for least chipmunks and Wyoming ground squirrels (Table 6). Additionally, white-tailed prairie dog detection probability was lower in areas with greater shrub cover, leporid detection probability was greater in areas with greater sage cover, and ground squirrel detection was greater in areas with more bare ground (Table 6, S2 Fig). All detection covariates were selected at the 1000-m scale.

### Model fit

The goodness of fit bootstrap procedure showed that the least chipmunk and leporid models appropriately fit the data (least chipmunk: *p* = 0.40; leporid: *p* = 0.27), while the Wyoming ground squirrel and white-tailed prairie dog models exhibited over-dispersion (Wyoming ground squirrel $\hat{c}=\;1.5$, white-tailed prairie dog $\hat{c}=\;1.4$, *p* < 0.01); therefore, we used the over-dispersion parameter to correct standard errors, coefficients, and predictions from these models, and QAIC_c_ for model selection (see Methods).

## Discussion

Our results demonstrate the efficacy of using remotely sensed data to estimate above-ground density for small mammals at both local and landscape scales, as well as the importance of native sagebrush and grassland habitat to their abundance. We showed that remotely sensed covariates and methods accounting for imperfect detection can be used to improve the fit of density models for a variety of common prey species in Wyoming. All species groups responded more strongly to measures of vegetation composition at the 1-km scale, indicating prey densities may be more dependent on the composition of the surrounding area than the habitat in their immediate vicinity. We also modeled density of each species group across the shrub-steppe and grassland habitat of Wyoming, providing the first extensive, spatially explicit depiction of above-ground prey availability in Wyoming, which can be used in a variety of conservation applications.

Herbaceous ground cover and bare ground were important predictors of density for white-tailed prairie dogs, least chipmunks, and Wyoming ground squirrels, and marginally predictive for leporids. These two covariates are inversely correlated (r > |0.82|), and therefore both describe a similar amount of ground cover. For least chipmunks, a low proportion of herbaceous cover had the single greatest positive effect on density, which corresponds with existing work showing a preference in least chipmunks for shrub-covered habitat [42]. The proportion of sagebrush or shrub cover within the larger landscape area of the sampled transects was a strong predictor of density for all four species groups, and was the top predictor for leporids, although this was a negative relationship for all but least chipmunks, indicating greater prey density in areas with relatively less sagebrush or shrubs. Previous work found similar correlations, with Wyoming ground squirrels shown to prefer open areas to closed shrub cover [67] and white-tailed prairie dogs frequently associated with sparse vegetation [68]. Among leporids, white-tailed jackrabbits are most commonly found in open areas such as grasslands or meadows and occur less often in sagebrush [53,69], and desert cottontails are found in a variety of habitats [52,69].

White-tailed prairie dogs were the most abundant species we modeled, followed by Wyoming ground squirrels, leporids, and least chipmunks (Table 3). Since we modeled above-ground density for all species, our estimates for semi-fossorial sciurid species were considerably lower than existing estimates in the literature that used trapping grids. For example, Clark [70] found between 21 and 118 Wyoming ground squirrels per km^2^ in grasslands near Laramie, Wyoming, while we predicted an average of 8.7 per km^2^ and a maximum site density of 56.8 per km^2^. Our above-ground estimates reflect the semi-fossorial nature of the species studied, which retreat to burrows when threatened by predators or in the course of behavioral thermoregulation [67]. Although our surveys were conducted during the early morning hours, when temperatures were cooler and animals more likely to be out foraging, some part of the population was likely underground during our surveys. Our estimates for semi-fossorial species are, therefore, most accurately defined as density for the percent of the population that is above-ground and available to avian predators at any given time. Our overall density estimate for leporids of 3.4 per km^2^ was also lower than other studies, but this may reflect a cyclic low of the population [71], as our estimates are more similar to population lows of 7 or 11.7 per km^2^ reported elsewhere [72,73].

Another factor in our low estimates is the large amount of environmental heterogeneity encompassed by the broad spatial extent at which we conducted our sampling [74,75]. Existing small mammal surveys have generally taken place in smaller study areas known to have populations of the species of interest. Conversely, our surveys took place at randomly sampled locations or near ferruginous hawk nests, neither of which were guaranteed to possess the habitat conditions required for a given small mammal species, resulting in relatively more sites with zero detections, and low average density over all sites. Although our approach may limit comparison of densities with smaller study areas that were not randomly selected, our results provide a characterization of prey density that is more relevant to the landscape-level ecology of highly-mobile species, like raptors, and to contemporary landscape-scale conservation planning.

Mapped above-ground density predictions based on our top models showed spatially distinct areas where each species group had its most dense distribution. Least chipmunks and Wyoming ground squirrels had greatest density in the southern area of the state, in the Wyoming Basin. This is a semiarid region of rolling shrub-steppe, dominated by Wyoming big sagebrush (*Artemisia tridentata*), and mixed with areas of salt desert and foothill shrublands [39]. Densities of Wyoming ground squirrels were greatest in the southeastern portion of the Wyoming Basin, whereas least chipmunks were most abundant in the southcentral portion. White-tailed prairie dogs showed a heterogeneous mixture of densities across their distribution, with areas of highest density in the Laramie and Shirley Basins and the sub-irrigated high valleys of the Green River and Saratoga Basins. The Wyoming Basin is heavily developed with oil and gas wells and has some of the highest potential for future development in the western United States [76]; thus, future habitat loss or fragmentation due to energy development may be in areas of high density for these species. Leporid species were most densely distributed in the north-central region of the state, in the Bighorn and Powder River Basins. The Bighorn Basin is lower in elevation, warmer, and drier than other parts of our study area and has greater prevalence of saltbrush-greasewood vegetation type, while the Powder River Basin is a semiarid prairie, dominated by mixed-grass vegetation [39].

The broader 1000-m scale was supported over the 250-m scale for the majority of variables, indicating that density of prey species groups we studied was tied to vegetative and topographic characteristics that occurred at a relatively broad spatial extent. This has implications for management to reduce loss and fragmentation of sagebrush and grassland habitat. Fragmentation due to roads associated with energy infrastructure can be intense; for example, the Big Piney/LaBarge oil field in Wyoming has between 3.1 and 5.3 km/km^2^ of road [30,77]. Our results suggest this level of fragmentation, even when native vegetation remains, may reduce the amount of contiguous vegetation within a 1-km radius enough to negatively impact density of some prey species. Habitat conversion, such as reduction of sage to improve land for grazing or encroachment of coniferous species, may negatively impact prey populations at a scale larger than the footprint of those areas that are converted. Our results support consideration of a spatial neighborhood of at least a 1-km radius to maintain prey density when modifying native habitat.

Destruction of native shrub habitat, whether by fire, removal of shrubs for agriculture, or conifer encroachment, generally results in the invasion of non-native species and a reduction in vegetation diversity [78,79]. Once lost, sagebrush communities and, to a lesser extent, native grassland communities, can be very difficult to restore [79]. Our study illustrates the importance of vegetation cover to small prey species, and therefore to their predators. Differing responses of species groups to vegetation indicate that changes in the amount or distribution of sagebrush or shrub communities will have species-specific effects. The negative response to shrub or sage cover and the positive response to herbaceous cover or bare ground by all species groups, except least chipmunks, indicates that more open habitat is favorable to these species. Native sagebrush and shrub habitat in the Wyoming Basin generally consists of multi-storied vegetation, with an overstory of shrubs and an understory of grasses and forbs [80]. The displacement of these communities with non-native plants [81–83], such as encroaching conifers or annual invasive grasses, may thus adversely affect these species.

## Supporting information

S1 Fig Modeled response of above ground density (individuals/km^2^) as a function of sampling occasion for each small mammal species group during surveys in Wyoming, 2010–2012. (TIF)

S2 Fig Modeled detection functions for each small mammal species group surveyed in Wyoming, 2010–2012, as influenced by occasion or vegetation. (TIF)

S1 Table Means and standard deviations of covariates used to model small mammal abundance in the sagebrush steppe and grassland regions of Wyoming, 2010–2012. (DOCX)
