# Supplementary figures and images for: Predicting above-ground density and distribution of small mammal prey species at large spatial scales
Source: PLoS One. 2017 May 17;12(5):e0177165. doi: 10.1371/journal.pone.0177165 (PMC5435308; doi:10.1371/journal.pone.0177165)

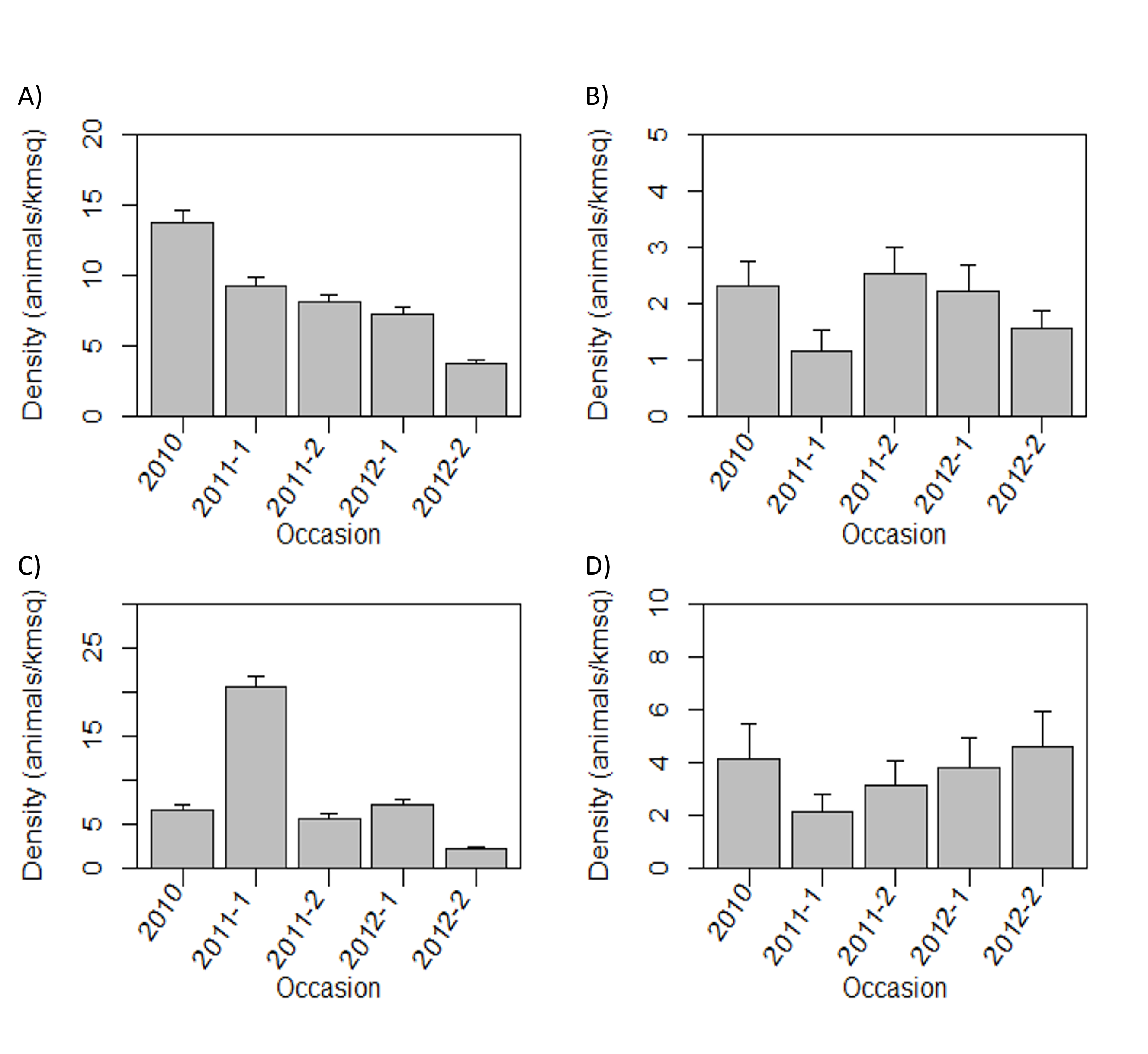

Supplement: S1 Fig — (TIF) [file pone.0177165.s001.tif]

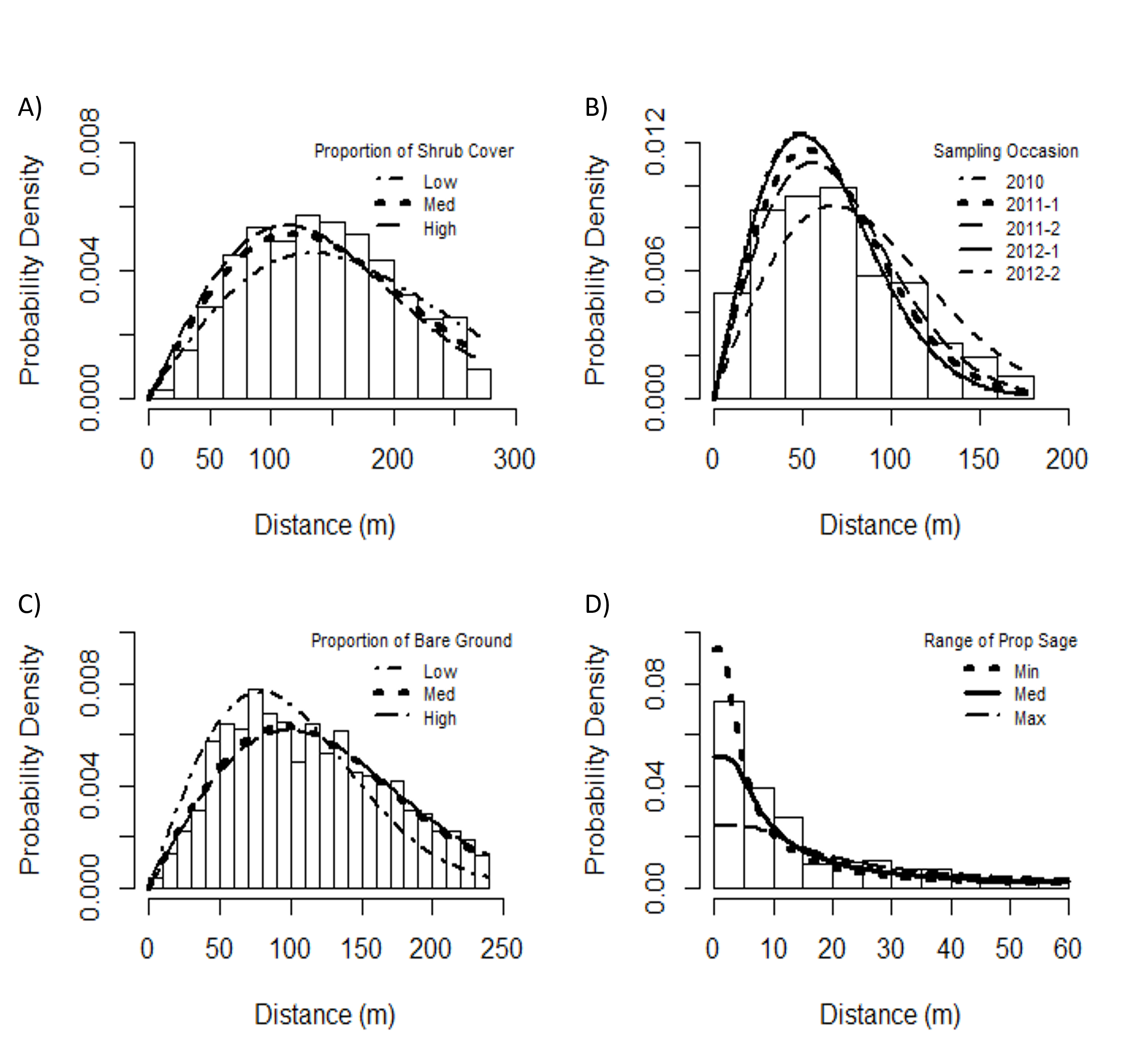

Supplement: S2 Fig — (TIF) [file pone.0177165.s002.tif]
