# Supplementary material for: Predicting above-ground density and distribution of small mammal prey species at large spatial scales
Source: PLoS One. 2017 May 17;12(5):e0177165. doi: 10.1371/journal.pone.0177165 (PMC5435308; doi:10.1371/journal.pone.0177165)
Supplement: S1 Table — (DOCX) [file pone.0177165.s003.docx]

| **S1 Table. Means and standard deviations used to standardize each covariate raster.** | | |
| --- | --- | --- |
| Covariate | Mean | Standard Deviation |
| Bare250 | 48.63 | 19.43 |
| Herb250 | 23.54 | 15.13 |
| Sage250 | 8.19 | 4.24 |
| Shrub250 | 9.79 | 4.58 |
| ShrubHt250 | 23.02 | 11.46 |
| Elevation250 | 1947.02 | 497.21 |
| TPI250 | 0.00 | 11.06 |
| Roughness250 | 919.16 | 39.22 |
| Productivity250 | 4306.74 | 1659.74 |
| Well_Dist | 41125.15 | 42212.07 |
| Rd_Dist | 1553.84 | 2064.30 |
| Ppt_Spring | 54.51 | 21.26 |
| Ppt_Winter | 29.96 | 32.37 |
| Temp_Spring | 13.50 | 5.24 |
| Tmax_Summer | 27.46 | 3.62 |
| Tmin_Winter | -11.77 | 2.09 |
| Bare1k | 47.82 | 18.70 |
| Herb1k | 24.04 | 14.66 |
| Sage1k | 8.22 | 3.89 |
| Shrub1k | 9.87 | 4.24 |
| ShrubHt1k | 23.43 | 11.43 |
| Elevation1k | 1946.62 | 495.67 |
| TPI1000 | 0.00 | 23.86 |
| Roughness1k | 919.17 | 33.12 |
| Productivity1000 | 4936.95 | 1726.05 |
